# Enhancing Beta Cell Replacement Therapies: Exploring Calcineurin Inhibitor-Sparing Immunosuppressive Regimens

**DOI:** 10.3389/ti.2023.11565

**Published:** 2023-06-08

**Authors:** Rossana Caldara, Valentina Tomajer, Lorenzo Piemonti

**Affiliations:** ^1^ Regenerative and Transplant Medicine Unit, IRCCS Ospedale San Raffaele, Milan, Italy; ^2^ Pancreatic Surgery, Pancreas Translational and Clinical Research Center, IRCCS Ospedale San Raffaele, Milan, Italy; ^3^ Diabetes Research Institute, IRCCS Ospedale San Raffaele, Milan, Italy; ^4^ Università Vita-Salute San Raffaele, Milan, Italy

**Keywords:** islet transplant, immunosuppression, calcineurin inhibitors, calcineurin inhibitor toxicity, tolerance induction

The study conducted by Wisel et al. [1] offers valuable insights into the long-term outcomes of beta cell replacement therapies and the use of immunosuppression in managing Type 1 diabetes (T1D). This 10-year follow-up study examined ten consecutive non-uremic patients with T1D who underwent islet transplantation. The patients were treated with calcineurin inhibitor (CNI)-sparing immunosuppressive regimens using either belatacept (BELA) or efalizumab (EFA). Out of the 10 patients, four achieved long-term insulin independence for an average duration of 13 years following a single islet infusion. On the other hand, six patients experienced failure of their initial islet transplant, with an average time to failure of 19 months. Four of these patients received a second islet infusion, one patient declined a second infusion and returned to insulin use, and one patient proceeded to undergo pancreas-after-islet (PAI) transplantation. Among the patients who received a second islet transplant, the average duration of insulin independence was 45.5 months. However, all four patients eventually reverted to insulin use. Two of them subsequently underwent PAI transplantation, while the remaining two continued to rely on exogenous insulin. At the time of publication, six out of ten patients still maintained insulin independence, including the three patients who underwent PAI transplantation.

The achievement of 40% insulin independence at 10 years following a single islet infusion is to be considered one of the most remarkable results ever reported. Comparatively, recent retrospective analyses using different immunosuppression regimens and multiple infusions have reported insulin independence rates of 4.8% [2], 20% [3], and 28% [4] at 10 years. Moreover, it significantly exceeds the prevalence of insulin independence reported in the Collaborative Islet Transplant Registry (CITR) 11th allograft report, where the value of 40% is achieved approximately 2 years after the last infusion [5]. Remarkably, this rate is closely comparable to the best outcome achieved after 10 years in solitary pancreas transplantation [6].

The authors aimed also to minimize the risk of nephrotoxicity associated with CNIs. Hence, an important question addressed in this paper is whether there was an advantage in preserving renal function. During the observational period of the study, there was a slight reduction in the estimated glomerular filtration rate (eGFR) from the initial value of 76.5 ± 23.1 mL/min, indicating an annual decline of 1.93 mL/min (1.76 and 2.1 mL/min in patients receiving BELA or EFA, respectively). This decline was minor among subjects with functional islet grafts (1.19 mL/min), higher in patients undergoing PAI transplantation with CNIs (3.45 mL/min). To assess whether these values indicate an advantage in preserving kidney function, we can compare them to two reference populations. The first population consists of an age-unadjusted cohort of 1,141 individuals with T1D who were followed in the Diabetes Control and Complications Trial (DCCT) and the Epidemiology of Diabetes Interventions and Complications (EDIC) study. This population started with a mean eGFR level of 126 mL/min. Over the 22-year duration of the study, an average decrease in eGFR of 1.2 and 1.56 mL/min per year was reported for the intensive therapy and conventional therapy groups, respectively [7]. The second reference population consists of an age-unadjusted cohort of 1,108 individuals with T1D who received islet transplantation alone and were followed by the CITR. This population started with a mean eGFR level of 91 mL/min. Over the 5-year period following the last infusion, they experienced a mean decline in eGFR of 2.4 mL/min per year [5]. Based on these comparisons, while the preservation of renal function in the study’s patients is not as favorable as that seen in the DCCT/EDIC cohorts, it does show an advantage over the decline observed in the CITR cohort. Further analysis and comparisons with larger matched reference populations would be beneficial for a more comprehensive assessment of the advantages in preserving kidney function with CNIs-free immunosuppression.

The involvement of CNIs in the NFAT signaling pathway, crucial for Treg differentiation, maintenance, and suppressive abilities, can have significant consequences [8]. It undermines immune tolerance and raises the risk of immune-related complications. Additionally, it diminishes the effectiveness of adoptive therapy that employs tolerogenic donor-specific Tregs. Hence, there is a requirement for research to examine the safety and feasibility of immunosuppressive regimens that minimize the utilization of CNIs [9, 10]. The study by Steven A. Wisel et al. highlights two significant findings: 1) patients treated with BELA showed stable levels of Tregs compared to circulating T cells in the first year after islet transplantation; 2) patients who received EFA exhibited increased levels of circulating Tregs, including a remarkable case with a substantial expansion of Tregs following islet transplantation. What’s truly remarkable is that even after discontinuing EFA treatment, this particular patient maintained insulin independence for a 10-year period without any notable immune response towards the transplanted islets, indicating the presence of operational tolerance.

Regrettably, there is currently no available guidance or formal consensus on the optimal or standard immunosuppressive strategy for human islet transplantation. Over the years, a significant shift in immunosuppression approaches has occurred in the absence of evidence-based practices (Figure 1). Several studies conducted on small cohorts have proposed various combinations of immunosuppressive agents [11–14]. These include T and B cells depleting agents (alemtuzumab, teplizumab, antithymocyte/lymphocyte globulin, rituximab), inhibitors of T-cell activation (IL2R antagonists daclizumab and basiliximab), replication inhibitors (azathioprine and mycophenolate mofetil/mycophenolic acid), mTor inhibitors (sirolimus and everolimus), lymphocyte tracking inhibitors (EFA), desensitizing agents (intravenous immunoglobulin), co-stimulation inhibitors (monoclonal antiCD28 belatacept/abatacept), CNIs (cyclosporine and tacrolimus), and anti-inflammatory agents (corticosteroids, IL1 receptor antagonist, and TNF-alpha inhibitors). It is important to note, however, that most of these studies were observational, predominantly retrospective or prospective single-center single-arm studies. Only one recently reported randomized controlled trial study focused on CXCR1/2 inhibitors stands out as an exception [15]. It is crucial to draw attention to the notable gap in consistent studies regarding the use of immunosuppression in the field of beta cell replacement, particularly considering the potential emergence of new sources of insulin-producing cells in the future. In this context, conducting research on immunosuppressive regimens that minimize the use of CNIs will greatly advance beta cell replacement therapies.

**FIGURE 1:**
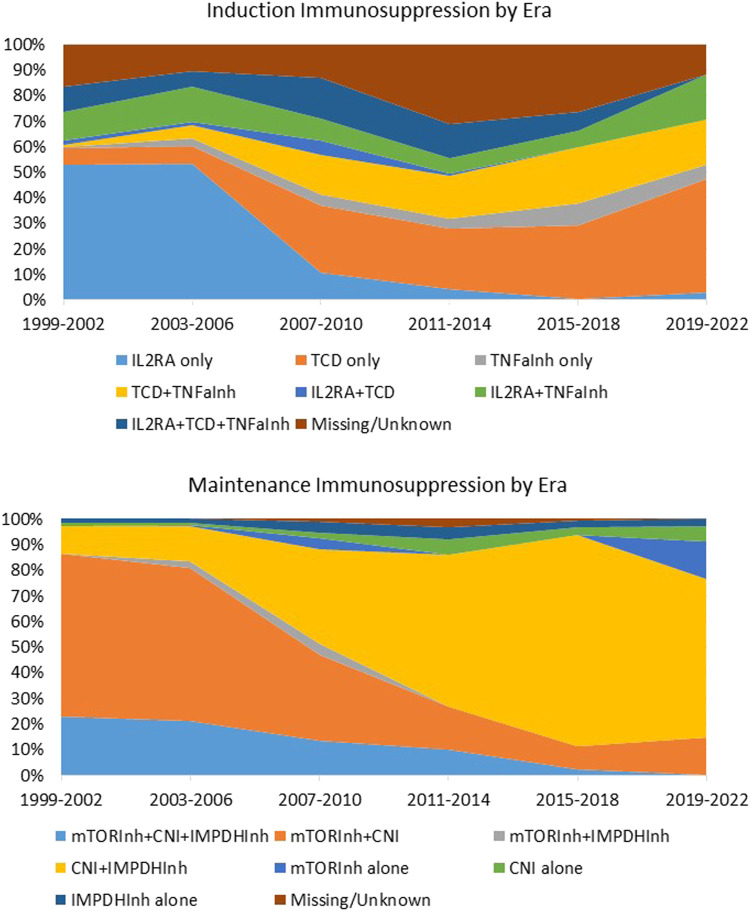
Induction and maintenance immunosuppression in islet transplantation by era. Immunosuppression regimen of 1,108 individuals with T1D who received Islet Transplant Alone (*n* = 992) or Islet after kidney (*n* = 186) between 1999 and 2022 and were followed by the CITR. Data source: Collaborative Islet Transplant Registry Coordinating Center: Eleventh allograft report 2022. TCD, T cell depleting agents; Inh, inhibitor; CNI, calcineurin Inhibitor; IMPDH, Inosine-5′-monophosphate dehydrogenase; IL1RA, IL1 receptor antagonist.

## Data Availability

Publicly available datasets were analyzed in this study. This data can be found here: https://citregistry.org/system/files/11th%20Allograft%20report%20May%2031%202022.pdf.
